# Supplementary material for: Difference between the Effects of Peripheral Sensory Nerve Electrical Stimulation on the Excitability of the Primary Motor Cortex: Examination of the Combinations of Stimulus Frequency and Duration
Source: Brain Sci. 2022 Nov 29;12(12):1637. doi: 10.3390/brainsci12121637 (PMC9775552; doi:10.3390/brainsci12121637)
Supplement: Supplementary file 1 [file brainsci-12-01637-s001.zip › brainsci-2053935-supplementary.pdf]

Supplementary Material

Supplementary Table S1. Experimental design and changes in MEP in participants before and after PES.

Participants	Sex	Frequency (Hz)	Time (min)	Pre-MEP (mV)	Post-MEP (mV)	MEP ratio
A	Female	10	40	0.30	0.56	1.83
		1	60	0.28	0.40	1.43
		50	20	0.15	0.15	1.00
B	Female	10	20	0.12	0.16	1.30
		50	60	0.46	0.65	1.42
		1	40	0.27	0.47	1.72
C	Female	1	20	0.13	0.12	0.88
		50	40	0.06	0.10	1.63
		10	60	0.05	0.05	0.96
D	Male	50	20	0.96	0.80	0.83
		10	40	0.59	0.72	1.23
		1	60	0.39	0.48	1.23
E	Female	1	20	0.37	0.63	1.71
		50	40	0.45	0.66	1.48
		10	60	0.18	0.30	1.72
F	Male	50	60	0.26	0.34	1.30
		1	40	0.16	0.18	1.15
		10	20	0.16	0.17	1.08
G	Female	50	60	0.21	0.36	1.71
		1	40	0.43	0.63	1.46
		10	20	0.20	0.24	1.22
H	Female	1	60	0.28	0.37	1.31
		50	20	0.18	0.17	0.93
		10	40	0.10	0.13	1.28
I	Female	10	60	0.12	0.25	2.17
		50	40	0.11	0.18	1.64
		1	20	0.23	0.28	1.20

J	Female	10	20	0.38	0.47	1.24
		50	60	0.28	0.30	1.06
		1	40	0.32	0.37	1.15
K	Female	1	60	0.77	0.99	1.29
		50	20	0.54	0.55	1.02
		10	40	0.59	0.96	1.63
L	Male	50	40	0.13	0.27	2.08
		10	20	0.07	0.10	1.29
		1	60	0.10	0.20	2.03
M	Female	10	40	0.11	0.17	1.55
		50	60	0.25	0.32	1.24
		1	20	0.19	0.19	0.99
N	Female	1	20	0.20	0.25	1.23
		50	60	0.67	1.10	1.63
		10	40	0.27	0.34	1.27
O	Female	50	40	1.25	1.29	1.03
		10	20	0.43	0.48	1.12
		1	60	0.36	0.45	1.25
P	Female	1	40	0.47	0.48	1.04
		10	20	0.43	0.53	1.25
		50	60	0.26	0.27	1.03
Q	Male	1	20	0.06	0.09	1.50
		50	40	0.15	0.18	1.23
		10	60	0.14	0.17	1.20
R	Female	10	60	0.14	0.26	1.86
		1	20	0.15	0.22	1.46
		50	40	0.16	0.22	1.45
S	Female	50	20	0.22	0.44	1.99
		10	60	0.57	0.68	1.20
		1	40	0.34	0.40	1.18
T	Female	10	40	0.06	0.10	1.57
		50	20	1.35	0.89	0.66

		1	60	1.02	1.12	1.10
U	Female	1	40	0.03	0.04	1.09
		10	60	0.26	0.37	1.40
		50	20	0.13	0.17	1.35

PES, peripheral sensory nerve electrical stimulation; MEP, motor evoked potential

Supplementary Table S2. MEP change and desirability before and after PES for each stimulus condition.

	Stimulus duration											
	20 min				40 min				60 min			
Stimulus frequency	Pre-MEP (mV)	Post-MEP (mV)	MEP ratio (CI)	Desirability	Pre-MEP (mV)	Post-MEP (mV)	MEP ratio (CI)	Desirability	Pre-MEP (mV)	Post-MEP (mV)	MEP ratio (CI)	Desirability
1 Hz	0.15± 0.07	0.19± 0.07	1.27 (1.08–1.47)	0.448	0.18± 0.09	0.20± 0.10	1.25 (1.06–1.45)	0.436	0.41± 0.21	0.44± 0.22	1.27 (1.08–1.47)	0.447
10 Hz	0.33 ± 0.12	0.40 ± 0.20	1.18 (0.99 –1.38)	0.401	0.26± 0.24	0.39± 0.39	1.49 (1.29–1.68)	0.560	0.28± 0.20	0.37± 0.22	1.46 (1.26–1.65)	0.545
50 Hz	0.56± 0.56	0.51± 0.3	1.03 (0.83–1.22)	0.325	0.42 ± 0.55	0.49 ± 0.53	1.45 (1.26–1.65)	0.540	0.37 ± 0.20	0.49 ± 0.40	1.34 (1.15–1.54)	0.483

Data are expressed as mean ± standard deviation.

CI, confidence interval; MEP, motor evoked potential
